# Evaluation of Recycled and Reused Metal Powders for DMLS 3D Printing †

**DOI:** 10.3390/ma17246184

**Published:** 2024-12-18

**Authors:** Simona Svozilova, Ivana Zetková, Juan Felipe Santa Marin, Jesús Arturo Torres Garay

**Affiliations:** 1 Faculty of Mechanical Engineering, University of West Bohemia, Univerzitni 8, 30614 Pilsen, Czech Republic; zetkova@fst.zcu.cz; 2 Tribology and Surfaces Group, Universidad Nacional de Colombia-Sede Medellín, Medellin 050034, Colombia; jfsanta@unal.edu.co (J.F.S.M.); jeatorresga@unal.edu.co (J.A.T.G.)

**Keywords:** additive manufacturing, MS1, DMLS, 3D printing, metal powders, recycling

## Abstract

Metal powders for additive manufacturing are expensive, and producing new ones from mined metals has a negative ecological impact. In this work, recycled and reused metal powders from MS1 steel for direct metal laser sintering (DMLS) 3D printing were evaluated in the laboratory. The powders were recycled by melting followed by gas atomizing. Virgin, recycled, and reused metal powders were evaluated using scanning electron microscopy (SEM), energy-dispersive X-ray spectroscopy (EDS), X-ray diffraction (XRD), metallography analysis, microhardness measurements, particle size distribution (PSD), shape factor by digital image processing (DIP), and flowability testing. The results showed that the particle distribution was modified after recycling. Kurtosis analysis revealed a reduction from −0.64 for virgin powders to −1.29 for recycled powders. The results demonstrated a positive skewness, indicating that the recycled powder contained a greater proportion of smaller particles. The shape factor was also modified and changed from 1.57 for virgin powders to 1.28 for recycled powders. The microstructure also changed, and austenite was found in the recycled powders. The microhardness of recycled powder decreased by 39% compared to the virgin powder. Recycled powders did not flow, using two different funnels to evaluate their flowability. The flowability of used powder was reduced from 4.3 s to 2.9 s.

## 1. Introduction

Metal additive manufacturing (AM) uses data from 3D models to fuse materials by applying layers repeatedly, creating the final structure. This method allows the development of geometrically complex objects that are difficult or impossible to produce using conventional methods, and it enables rapid prototyping. In the field of metal AM, there has been a rapid advancement in 3D printing technology, with advanced methods and innovative equipment. At the same time, the number of metal alloys that can be reliably printed continues to grow, providing opportunities for further research [1].

Metal components are the foundation of modern industries, such as aerospace, automotive manufacturing, and energy production. AM is a key strategic technology for innovation and also plays a role in industrial sustainability [2]. One of the inherent advantages of metal AM is that it generates less waste compared to conventional manufacturing, as material is added layer by layer to create an object. However, some waste still arises in the form of support structures and excess powder [3]. After printing, the amount of residual powder can be up to 40 wt.%, of which up to 10 wt.% of the original material cannot be reused. Sustainable practices, including recycling metal powder, are required to minimise residues.

Recycling and sieving increase material utilisation while making the process more sustainable. By incorporating material recycling, the demand for purchasing new raw materials, as well as the need for mining and processing mineral resources, is reduced. Reducing waste and maximising material usage are currently among the key challenges in many manufacturing processes aimed at minimising the environmental impact of industry. However, it is essential to consider the environmental impact of the recycling process itself, compared to standard production from new raw materials. A critical factor is how recycling or reuse affects the material properties and, consequently, the final part’s performance. Additionally, optimising this process to ensure the output material meets or exceeds required specifications is crucial [4].

Metal powders are commonly used in direct metal laser sintering (DMLS). Powder metallurgy industries commonly characterise metal powders by their chemical and physical properties. These powder characteristics significantly affect the quality and properties of the produced parts. Important factors include powder flowability, shape, morphology, particle size distribution (PSD), chemical composition, and powder density. These properties are crucial for ensuring consistent, repeatable, and validated additive manufacturing [5].

The recycling and reuse of metal powders is becoming an increasingly significant area of focus within the field of AM, with the objective of reducing both the financial costs associated with production and the environmental impact of the technology. Nevertheless, the reuse of metal powders in processes such as DMLS can result in alterations to powder characteristics that may impact the quality and mechanical integrity of the final printed components. The mechanical properties of Ti-6Al-4V components produced by selective laser melting (SLM) can vary with powder reuse, thus underscoring the necessity for consistent monitoring in order to maintain performance standards [6]. Another study [6] investigated the effects of powder characteristics on the reliability and performance of medical-grade components produced through selective laser sintering (SLS). Their findings demonstrated that changes in powder characteristics over multiple uses can influence the reliability and performance of these components [7]. Similarly, other researchers have shown that powder recycling in the laser powder bed fusion of 17-4 PH stainless steel can affect the quality of the printed part [8]. These studies highlight the need for optimised recycling protocols that ensure consistency in key material characteristics.

Additionally, the microstructural and mechanical properties of maraging steels manufactured with recycled powders were examined, demonstrating the potential of such materials to meet industrial standards when subjected to careful monitoring. These studies collectively emphasise that while powder recycling can significantly advance the sustainability of AM processes, it is crucial to assess and optimise the properties of each powder in the reuse cycle in order to prevent detrimental effects on product quality [9]. This paper builds on these findings by evaluating the performance and consistency of recycled metal powders in DMLS, focusing on material stability, mechanical properties, and the overall feasibility of implementing a systematic recycling approach.

From the literature review, papers with results relating to the characterisation of recycled powder from MS1 steel for DMLS were not found. Accordingly, the aim of this paper is to evaluate recycled and reused metal powders from MS1 steel for 3D DMLS. The powders were recycled by melting followed by gas atomising. Virgin, recycled, and reused metal powders were evaluated using scanning electron microscopy (SEM), energy-dispersive X-ray spectroscopy (EDS), X-ray diffraction (XRD), metallography analysis, microhardness measurements, particle size distribution (PSD), roundness shape factor by digital image processing (DIP), and flowability testing. The evaluation of recycled metal powders is relevant since the changes in the powder properties can affect not only the quality of the final products but also the long-term economic and environmental sustainability of the entire process [10].

## 2. Materials and Methods

### 2.1. Materials

All experiments and analyses were conducted on a metal powder called maraging steel (MS1). This material was developed specifically for AM by EOS (Krailling, Germany). It is designed for metal AM systems equipped with a 400 W laser source with a wavelength between 1060 and 1100 nm [11]. The deposited material is a maraging steel, supplied in the form of metal powder consisting of spherical particles. Its chemical composition corresponds to the U.S. classification 18% Ni Maraging 300, the European standard 1.2709, and the German X3NiCoMoTi 18-9-5 [12].

The MS1 steel has been widely used as tool steel. It has been used in the manufacturing of tools for injection moulding, die casting, and punching. After 3D printing, the material can be heat-treated to achieve hardness levels exceeding 50 HRC, making it suitable for high-volume production tools. The automotive industry requires precision and reliability, and MS1 steel is used in moulds for sheet metal forming. This work focused on MS1 since there is ongoing cooperation with some automotive companies to improve their forming processes.

The chemical composition reported by the manufacturer is shown in Table 1. The particle size range provided by the manufacturer is from 10 μm to 63 μm, with an average particle size of 50 μm.

In this work, three types of this material were compared: virgin (V), used (U), and recycled (R). The first sample, marked as V (virgin), is a material sourced directly from the supplier. The used powder (U) is material that has already been employed in the 3D printing process, then sieved using a 63 µm mesh by a sieving device and designated for reuse. The particles that remained on the sieve after sieving were collected and subjected to a recycling process, resulting in the final sample, R. Subsequently, an EOS M 100 (EOS GmbH, Krailling, Germany) printer was employed to assess the material’s suitability at the Faculty of Engineering from the University of West Bohemia. The recycled samples were created by melting the waste in a horizontal plasma furnace. This process produced a rod, which was then transformed into spherical particles for 3D printing by gaseous atomisation (argon), as in the case of EOS production.

Gas atomisation (GA) is a metal powder production technique that involves the disintegration of molten metal into fine droplets using high-velocity jets of inert gas. This process results in the rapid solidification of the droplets into spherical powder particles, which typically range in size from 10 to 150 microns. Another method used to recycle powder is ultrasonic atomisation (UA). There are several differences related to the energy consumption and cost efficiency of GA when compared to UA. The most important factors are the quality of the produced powders, production rates, and operational costs [13]. For GA, a significant amount of energy is required to maintain high gas pressures (between 0.7 and 6 MPa) for atomizing molten metal. Energy consumption is influenced by the need for a controlled atmosphere to prevent oxidation, which can further increase operational costs due to gas purification systems. UA uses ultrasonic vibrations to create fine droplets from molten metal. This method typically consumes less energy than GA because it operates at lower pressures and temperatures. The ultrasonic system can achieve high conversion rates (>95% of raw material into usable powder) with relatively lower energy input compared to high-pressure gas systems.

However, GA is widely used in industry, but it involves higher operational costs due to energy consumption, the maintenance of gas systems, and the need for extensive safety measures against oxidation. The initial setup costs for GA facilities can also be significant, which may impact overall cost efficiency in low-volume production scenarios. UA is emerging as a more cost-effective alternative, particularly for small-scale production. Although the mass output of UA may be lower than that of GA, the quality of the powders produced (finer particle size, higher sphericity, and better flowability) can lead to reduced waste and improved performance in applications like additive manufacturing. This can translate into savings in material costs and enhanced productivity in downstream processes.

In this work, the aim was to evaluate recycled powder by gas atomisation. Ultrasonic atomisation was not available to perform tests, and on the other hand, the aim was not to obtain a finer average particle size and narrower distribution, as expected for UA. In addition, a minimum of 10 kg of recycled powders was required for the tests, but the production rate of UA did not allow for obtaining these quantities.

### 2.2. Laboratory Testing

The properties of the final product made from general metal powders depend on the characterisation of the powder during production. The main characteristics that describe metal powders are as follows: chemical composition, particle size distribution and shape, apparent density, and flowability. All three samples were subjected to various analyses and experiments under the same parameters. The tests included all the aforementioned properties, except for apparent density. Additionally, two more analyses were conducted: phase analysis of the particles and the measurement of the microhardness of individual particles.

One of the methods used was SEM. SEM images were taken to analyse changes in the external morphology of the particles and to evaluate the chemical composition of the samples. EDS was used to identify and quantify the elements present in a sample. The JEOL JSM 5910LV (JEOL, Akishima, Japan) was employed to obtain several images and conduct EDS analyses on the three powders. The JEOL JSM7100F (JEOL, Akishima, Japan) was used to acquire high-resolution images. The samples were not coated with sputtered gold and were mounted directly onto carbon tape. The results from these devices were then evaluated using digital image processing (DIP), where SEM images were analysed for particle size distribution and shape factor using ImageJ software (1.54g. Wayne Rasband anc contributors. National Institutes of Health, USA).

Additionally, XRD was used to identify the phases and the crystalline structure of the materials. The diffraction patterns were obtained using a PANalytical diffractometer with a Pixel 3D detector, operated with Cu Kα radiation (λ = 1.5403). An omega/2-theta goniometer was used, and the platform configuration included a spinner operating at 4 revolutions per minute (rpm). The time per step was 52 s and the measurement step size was 0.05°. Measurements were made using a voltage of 45 kV and a current of 40 mA.

The internal structure of the powder was analysed using a cold mounting method. The powder samples were mixed using a resin and hardener to obtain cylindrical samples. After mounting, the samples were polished using emery papers and a cross-section of the particles was analysed using light optical microscopy (LOM) after etching with a prepared solution of Kalling’s No. 2 (CuCl_2_, hydrochloric acid (HCl), and ethanol mix). The microhardness of the individual particles was measured using a microhardness tester. Each sample was measured eight times in an Indentec zhl microhardness tester by applying a 25 g load for 10 s, according to ASTM E384, using the Vickers formula:

(1) $$\left(\frac{1854\times P}{D^{2}}\right)$$

The particle size distribution was obtained using a Mastersizer 2000 (Malvern Panalytical B.V., Lelyweg 1, 7602EA Almelo, The Netherlands) device and the dynamic light scattering (DLS) method. The device measures particle size using a laser, with the particles flowing in a liquid, in this case, water.

One of the essential properties of the powder is its flowability, as described by the ASTM B213-20 standard [14]. This test method includes the determination of the flow of metal powders and powder mixtures using a Hall flowmeter funnel. It is only suitable for powders that flow through the funnel without assistance. For this experiment, a funnel according to the standard was used. The first calibration measurement was performed using distilled water and copper powder (98% Cu). Since the recycled powder did not flow using the standard Hall flowmeter, a second and third test were performed using a funnel with angles of 15° and 1.42°, made of polypropylene. The objective of the study was also to assess the impact of powder recycling on the tensile strength and fatigue resistance of printed parts. However, the recycling process encountered difficulties when attempting to print with the DMLS printing machine EOS M100 (EOS GmbH, Krailling, Germany). Specifically, the powder particles did not flow as intended, and the powder was not distributed evenly, resulting in a non-uniform layer on the build platform.

## 3. Results

This section presents the results of a comparison of the impact of virgin (V), sieved (U), and recycled (R) metal powders on their properties for additive manufacturing. The testing focused on analysing these three types of powders, monitoring changes in particle size, shape, chemical composition, phases of individual particles, microhardness, grain structure, and flowability. The objective was to evaluate how recycling affects the quality of the powder compared to the virgin and used (sieved) materials.

### 3.1. Morphology

**Figure 1:**
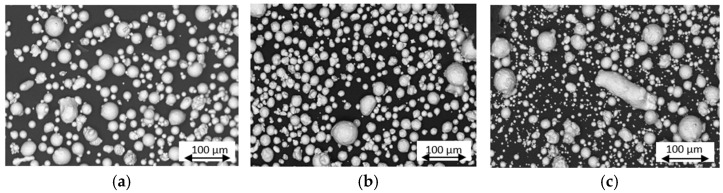
Scanning electron microscopy (SEM) images of MS1 powder: (**a**) virgin, (**b**) used, (**c**) recycled.

Figure 1 shows low-magnification micrographs of the three types of powders evaluated. A notable difference in particle size distribution was observed among the three types of powders: virgin, used, and recycled. Recycled powders exhibited a higher number of smaller particles along with greater variability in particle size distribution.

**Figure 2:**
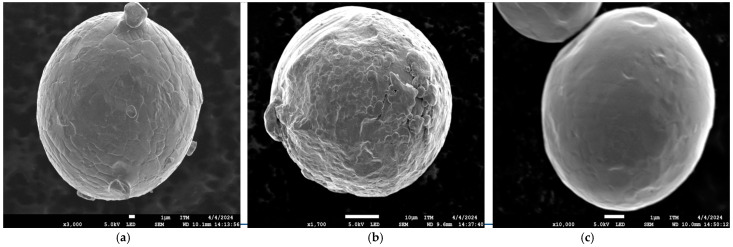
SEM images of particles of MS1 powder: (**a**) virgin, (**b**) used, (**c**) recycled.

Figure 2 shows high magnification micrographs of individual particles. Virgin and used powders exhibit “satellites” on their surfaces, whereas recycled particles have a smoother surface. Two types of satellites were identified: adhered and fused. Adhered satellites might result from electrostatic forces exerted by larger particles on smaller ones. On the other hand, fused satellites could originate from particle agglomerations during the atomisation process or from interaction with the laser during the printing process. Furthermore, recycled powders exhibited a higher presence of agglomerates composed of multiple particles of varying sizes.

### 3.2. Particle Size Distribution

**Figure 3:**
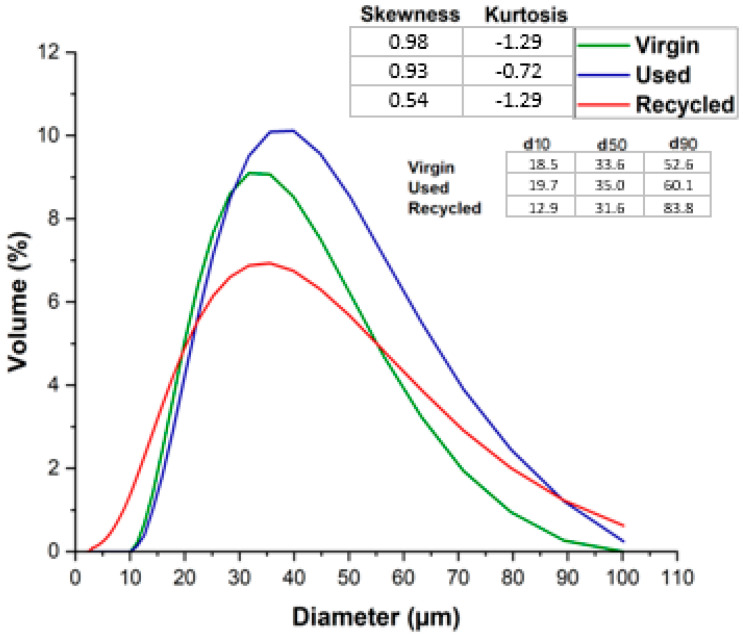
Particle size distribution histogram—DLS results.

Figure 3 shows the results related to particle size distribution using DLS. Virgin powder has the highest proportion of particles, between 30 and 35 µm. The used powder has the highest proportion of particles, between 35 and 40 µm and at larger scale than the virgin or recycled materials. Virgin and used powders show values very similar to those reported by the manufacturer. The graph illustrates an increase in recycled powder at lower values, comprising grains with a diameter of approximately 30 µm, with a notable degree of dispersion in the values.

The kurtosis results for each powder type reveal a reduction in both used and recycled powders compared to the virgin powder. This suggests a broader distribution of particle sizes, particularly more pronounced in the recycled powder. Notably, the used powder exhibits only a marginal variation in kurtosis and skewness relative to the virgin powder, while the recycled powder shows a significant shift. Additionally, all powders display a positive skewness, indicating a higher proportion of particles smaller than the mean size compared to those larger than the mean.

### 3.3. Shape Factor from SEM Images

**Figure 4:**
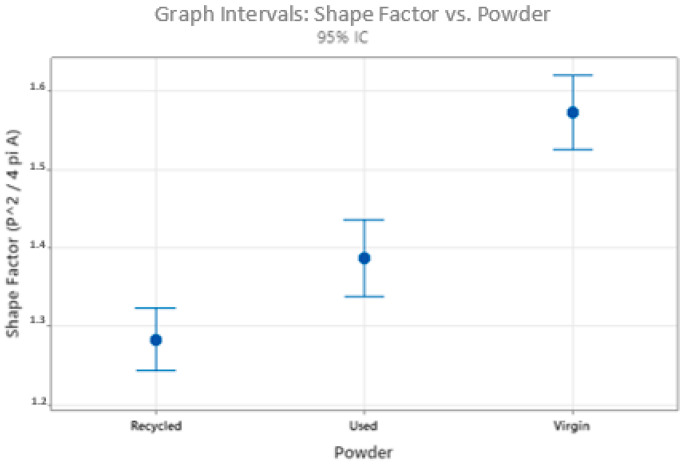
Roundness shape factor of particles after digital image processing from SEM images.

Figure 4 presents the results associated with the average roundness shape factor (P^2/4 pi A) for each type of powder. The ANOVA results indicate that there is statistically significant evidence to conclude that the average shape factor values differ among the three types of powders. Recycled powders show a greater degree of roundness. In the following, the results of microstructural analysis are presented, which show that remelting affects the internal structure of the grains. The results of the circularity can also affect the atomization parameters, including pressure, distance of grain impact from the nozzle, and flow rate.

### 3.4. Chemical Composition

Table 2 shows the chemical composition of the three analysed powders. A change was not expected during the recycling process. However, the samples showed potential contamination. Carbon was excluded from the evaluation since the table was obtained by EDS analysis.

The results for virgin and used powder were not significantly different from the values given in the material sheet. The virgin powder was found to have a higher cobalt content, which may be attributed to possible contamination during the production or handling of the powder. The values for the used powder were in line with the expected values from the material data sheet.

In contrast, the recycled powder exhibited changes in chemical composition. An increase in nickel and chromium content was observed, while titanium and molybdenum, which were present in the virgin and used powder, were entirely absent. Additionally, a decrease in cobalt content was noted compared to the virgin and sieved powders. These deviations may be due to contamination during the recycling process or chemical changes that occur during the recycling process itself. The atomisation gas was argon, used to obtain an inert atmosphere. On the other hand, since the process was performed by an independent company, they did not provide the purity of the gas.

### 3.5. Microstructure

**Figure 5:**
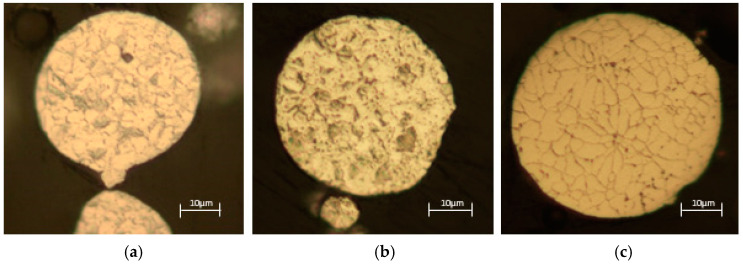
Typical microstructure of particles: (**a**) virgin, (**b**) used, (**c**) recycled.

Figure 5 shows typical examples of particles from the three different analysed powders. The results show that a changed microstructure was observed in the recycled powder. The microstructure of the virgin and used powders is similar and corresponds to martensite. The recycled powder has a microstructure with austenite grains and precipitates at the grain boundaries. The presence of austenite can significantly alter the mechanical properties of the recycled powders, as austenite is softer than martensite. The occurrence of austenite may be due to the heat treatment these powders undergo during the specific recycling processes (gas atomisation).

**Figure 6:**
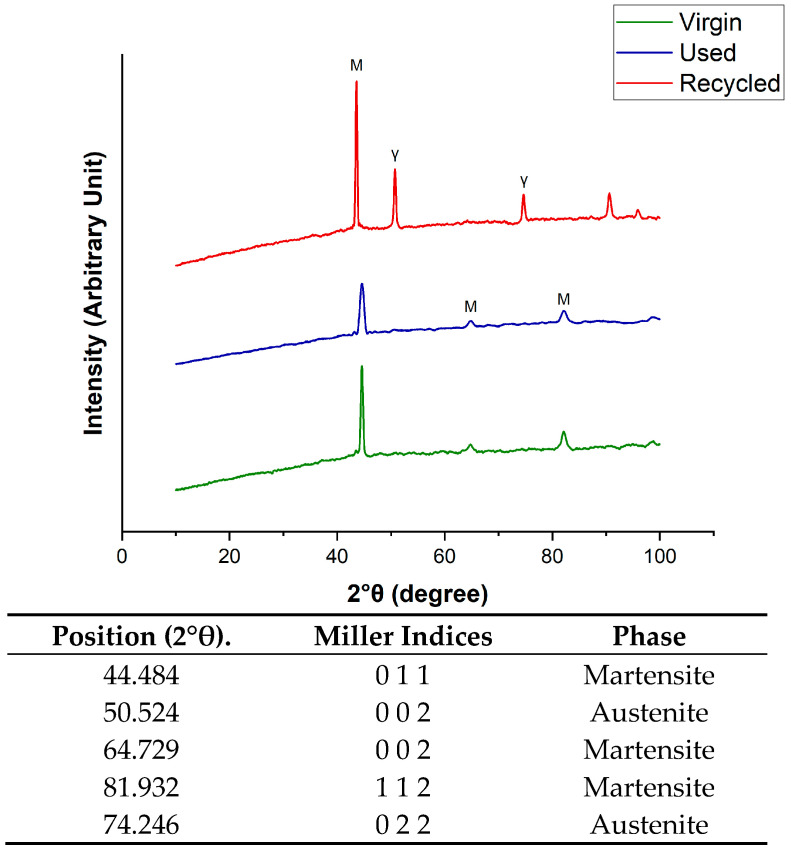
XRD patterns of evaluated powders.

Figure 6 shows the XRD patterns of the evaluated powders. The virgin and used powders exhibit the same phases, both characteristic of martensite, at positions 44.484, 64.729, and 81.932. However, the recycled powders show two peaks associated with austenite at positions 50.524 and 74.246.

### 3.6. Microhardness

**Figure 7:**
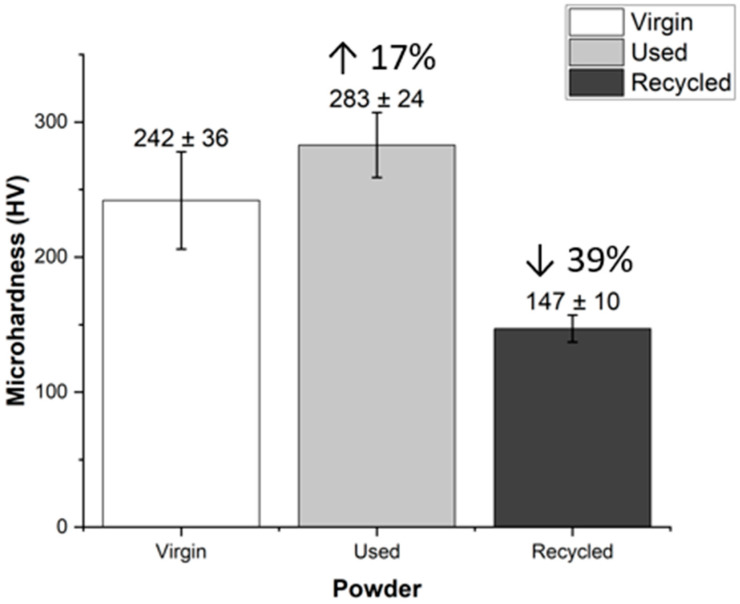
Microhardness of powders.

The obtained microhardness values are particularly noteworthy. The virgin powder exhibited a microhardness value of 242 ± 36 HV. In the case of the reused powder, there was a 17% increase in microhardness to 283 ± 24 HV, likely due to material hardening because of laser exposure during the printing process. Conversely, the microhardness within the grains of the recycled powder decreased by nearly 39% compared to the sieved powder, dropping to 147 ± 10 HV. Figure 7 shows a graph with the results of the microhardness testing.

These results show that changes in the microstructure of the powder particles lead to a reduction in microhardness due to the formation of austenite. From the microstructural analysis, austenite and precipitates were also found. The initial powder has martensite, which is harder than austenite. After recycling, austenite is formed with some precipitates, and consequently, the hardness is reduced. Accordingly, the observed drop in hardness is related to the phase transformation in recycled powders. The observed changes in microhardness have implications for the mechanical properties of the final product, making their monitoring crucial. These findings demonstrate how recycling and powder processing can impact the quality and performance of additively manufactured parts.

Heat treatment significantly affects the mechanical properties of MS1 (maraging steel) powders, enhancing their performance for various applications. The primary heat treatment processes include solution annealing followed by aging, which together optimize the microstructure and mechanical characteristics of the material. A typical process to increase hardness is aging. Aging is performed in MS1 steel after solution annealing and subsequent heating at temperatures typically between 480 °C and 510 °C for several hours. Aging leads to precipitation hardening, where fine intermetallic compounds form within the matrix. Future work for recycling MS1 steel powder could be related to using aging to restore the mechanical properties of recycled powders. Another interesting evaluation would be to reduce the flow rates during gas atomisation to avoid producing finer powders and improve yield by enhancing interaction between the gas and moulded metal.

### 3.7. Flowability

**Figure 8:**
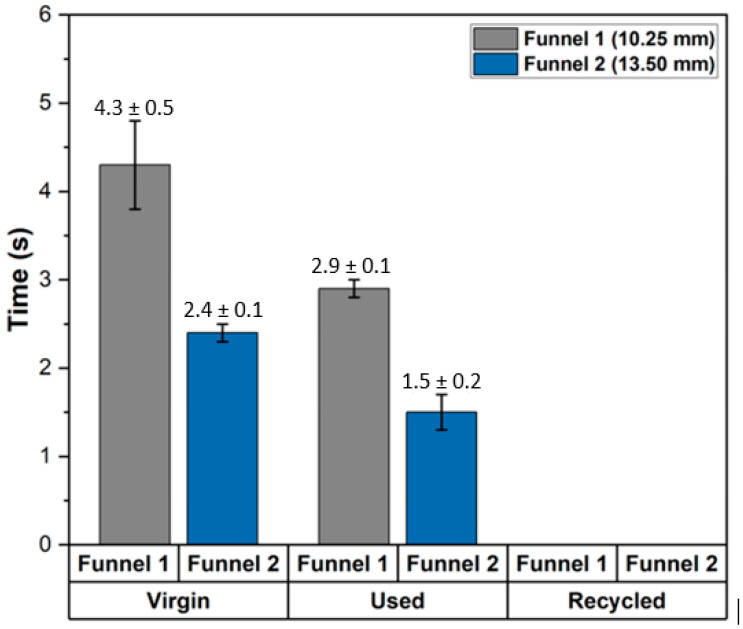
Flowability of powders.

The MS1 metal powder was not suitable for flowability measurements using the Hall flow meter. Consequently, two alternative measuring systems were employed: a plastic funnel with a bottom diameter of 10.25 mm and another with a diameter of 13.45 mm. During the test, the flow time and the weight of the powder passing through the funnel were recorded. However, it was not possible to obtain results for the recycled powder, even when the larger bore funnel (13.45 mm) was used. These tests were conducted solely to compare the flowability of the three powder samples: new, used, and recycled. The results showed that the flowability of the used powder is more than 30% lower than the virgin powder. Figure 8 shows a graph with the results of the flowability tests.

## 4. Discussion

The results of this study revealed significant differences in the properties of virgin, used, and recycled metal powders, which have a substantial impact on their use in additive manufacturing. The findings demonstrate how recycling affects key powder properties such as flowability, microhardness, particle size distribution, roundness shape factor, and microstructure, all of which can directly influence the quality of 3D-printed parts.

Particle size distribution and shape factor analysis revealed that the recycled powder had a broader particle size distribution, particularly with an increased quantity of smaller particles. Additionally, the recycled powder had a less uniform shape compared to the virgin and used powders. These differences can affect not only flowability but also the powder’s ability to spread evenly, further complicating the printing process.

Flowability tests showed that the recycled powder exhibited significantly poorer performance due to the presence of smaller particles, which hindered the powder’s free flow. This result aligns with previous knowledge, indicating that particle size distribution plays a crucial role in determining powder flowability. A higher proportion of smaller particles not only reduces flowability but may also affect the powder’s ability to form uniform layers during printing, potentially leading to defects in the final product. Flowability measurements were performed using alternative settings due to the non-functionality of the standard Hall flowmeter for the samples analysed. The use of non-standard pipettes with different diameters introduced variability, especially for the recycled powder, where finer particles prevented accurate flow measurement. This limited direct comparison of the three powder samples in terms of flow behaviour.

Several critical parameters must be carefully controlled to achieve particle uniformity in gas atomisation. These parameters directly influence the size, shape, and distribution of the metal powders produced. Gas atomisation has some key parameters that must be controlled. The atomisation gas pressure typically ranges from 2 to 10 MPa. Higher gas pressure results in finer particles due to increased energy imparted to the molten metal stream, which enhances atomisation efficiency and reduces particle size distribution. The gas flow rate is crucial, as it influences the cooling rate and droplet size. Higher flow rates can produce finer powders and improve yield by enhancing interaction between the gas and molten metal. The temperature of the molten metal before atomisation is also important, since the increment can reduce satellite formation (small particles that do not contribute to uniformity) and improve flowability, leading to a more uniform particle size. Other important parameters are the nozzle design, separation distance, gas to metal ratio, among others.

The microhardness of the recycled powder was 39% lower than that of the virgin powder. This reduction is attributed to changes in the microstructure of the powder during the recycling process, which led to the formation of austenite. The lower microhardness could compromise the mechanical properties of 3D-printed components made from recycled powder, raising concerns about their structural integrity. Microstructural analysis using XRD and LOM provided valuable information about crystalline phases and grain structure. Recycled powders showed changes in microstructure, which may explain the decrease in microhardness. The presence of new chromium-rich precipitates may affect the corrosion resistance of the deposited material.

Chemical composition analysis using SEM-EDS showed consistency between the virgin and used powders, with no significant deviations from the material specifications. However, the recycled powder showed potential signs of contamination or changes in composition, likely caused by the recycling process.

The reuse and recycling of powder is introduced as a means of reducing environmental impact. One of the most significant benefits is the reduction in the quantity of new material required, which consequently leads to a reduction in the associated mining operations. The selected recycling process entails the remelting of the material, which is energy-intensive and necessitates a substantial input of waste to be environmentally viable. In the context of this study, the laboratory does not generate the requisite quantity of waste for recycling, necessitating an investigation and adjustment of this process to align with the supplied quantity from a sustainability perspective. Alternative options include a comparison of other treatments for the waste powder or the selection of an alternative recycling process.

The environmental impact of recycling powders is an important topic. However, the data required for a comprehensive analysis, including energy consumption, costs, and CO_2_ emissions, are proprietary and are not available from the powder manufacturers. Consequently, the analysis could not be conducted due to a lack of information and the inability to make comparisons. The company supplying the virgin powder has its own expertise in production, which further complicates the analysis. In the case of GA, the precise details of the process are unclear. Consequently, one of the next steps of this study is to gain a deeper understanding of the process and identify potential contamination or the effect of GA parameters on powder characteristics.

This study demonstrates that recycling metal powders is a promising approach to reducing waste and material costs in additive manufacturing, but it also presents challenges that must be addressed. The degradation in the flowability, microhardness, and microstructure of recycled powders highlights the importance of optimising recycling processes and testing protocols to ensure that recycled powders meet the required performance standards for 3D printing.

The thermal history and potential contamination during remelting likely contribute to the degradation of powder quality, resulting in the alterations in microhardness, grain structure, and particle size distribution. Therefore, it is recommended to perform further studies focused on optimising the recycling process to mitigate these adverse effects and improve the performance of recycled powders in 3D printing applications.

## 5. Conclusions

The laboratory evaluations of virgin, used, and recycled powders of MS1 steel indicate that the properties of recycled powders for 3D printing using DMLS are decreased in comparison to both the properties of the virgin and used samples. Generally speaking, used powders showed better results compared to recycled powders.

The recycling process changed the particle size distribution from the highest proportion of around 35 µm to 30 µm. Smaller particles (less than 10 µm) were found in recycled powders.

The shape factor of recycled powders was also modified from 1.57 (virgin) to 1.28 (recycled) for recycled powders.

The microhardness of recycled powder was also reduced 39% to 147 HV. The reduction was caused by a microstructural change from martensite (virgin powder) to austinite and precipitates (recycled powder).

The flowability of used powder was reduced around 30%. Recycled powder did not flow through three different funnels. The limited flowability was caused by a large proportion of smaller particles that tend to agglomerate, preventing the powder from flowing properly.

Finally, it is important to evaluate and optimize recycling parameters such as atomisation pressure and sieving to obtain a narrow distribution of particles.

## Figures and Tables

**Table 1:** Chemical composition of MS1 powder reported by the manufacturer [11].

	Fe	Ni	Co	Mo	Ti	Al	Cr	Cu	C	Mn	Si	P	S
MS1	Bal.	17–19	8.5–9.5	4.5–5.2	0.6–0.8	0.05–0.15	0.5	0.5	0.03	0.1	0.1	0.01	0.1

**Table 2:** Chemical composition—EDS results.

	Fe	Ni	Co	Mo	Ti	Al	Cr	Cu	C	Mn	Si	P	S	O
MS1	Bal.	17–19	8.5–9.5	4.5–5.2	0.6–0.8	0.05–0.15	0.5	0.5	0.03	0.1	0.1	0.01	0.1	-
Virgin	65.8 ± 0.6	18.7 ± 1.2	10.3 ± 0.6	4.4 ± 0.6	0.6 ± 0.1	-	-	-	-	-	-	-	-	-
Used	65.8 ± 1.0	19.3 ± 0.4	9.5 ± 0.4	4.8 ± 0.8	0.6 ± 0.3	-	-	-	-	-	-	-	-	-
Recycled	46.6 ± 1.2	25.9 ± 3.6	6.3 ± 0.2	11.8 ± 1.2	-	-	3.8 ± 0.3	-	-	-	-	-	-	5.9 ± 2.0

## Data Availability

The original contributions presented in the study are included in the article, further inquiries can be directed to the corresponding author.
